# Acute Limb Ischemia Complicated by Heparin-Induced Thrombocytopenia in an Asymptomatic COVID-19 Patient

**DOI:** 10.7759/cureus.16162

**Published:** 2021-07-04

**Authors:** Nabeel A Siddiqui, Enkhmaa Luvsannyam, Molly S Jain, Muhammad Abbas, Arathi Jayaraman, Redjon Zhuleku, Nayaab Ullah, Alma Corona, Mohammad T Hussain

**Affiliations:** 1 Research, California Institute of Behavioral Neurosciences & Psychology, Fairfield, USA; 2 Medicine, Saint James School of Medicine, Park Ridge, USA; 3 Medicine, Avalon University School of Medicine, Willemstad, CUW; 4 Medicine, Xavier University School of Medicine, Oranjestad, ABW; 5 Medicine, Medical University of Lublin, Lublin, POL; 6 Hematology and Oncology, Windsor University School of Medicine, Cayon, KNA; 7 Hematology and Oncology, Advanced Cancer Care Center, North Aurora, USA

**Keywords:** acute limb ischemia, anticoagulation, coronavirus, covid-19, heparin-induced thrombocytopenia, rutherford classifications

## Abstract

Acute limb ischemia (ALI) is the sudden decrease in limb perfusion caused by embolism secondary to many blood stasis conditions. Treatment commences with intravenous (IV) unfractionated heparin infusion. Individuals can have an immune-mediated reaction to heparin products which results in heparin-induced thrombocytopenia (HIT). Coronavirus disease 2019 (COVID-19) has added to the difficulty of treating patients with ALI due to increasing the likelihood of HIT via the virus's ability to manipulate the coagulation parameters. We present a case of ALI complicated by HIT in a 49-year-old male with a confirmed asymptomatic COVID-19. The patient initially presented with progressive pain, coldness, and tingling in the right foot. He was treated with a tissue plasminogen activator (TPA) and a heparin drip. The occlusion persisted despite medical intervention leading to right below-knee amputation. The patient returned to the emergency department (ED) 13 days later with massive intracranial hemorrhage and subsequently expired. This case study demonstrates the significance of COVID-19 diagnostic testing due to the possibility of developing blood clots and potential complications, including HIT.

## Introduction

Acute limb ischemia (ALI) is defined as the sudden decrease in limb perfusion that can compromise limb function and can even result in life-threatening complications [1]. Studies report ALI incidence to be 1.5 cases out of 10,000 people every year [2]. The estimated mortality rate is 15%-20% from the acute onset due to underlying conditions as cardiovascular, cerebrovascular, and ischemia-reperfusion injury [2]. The primary causes of ALI include atrial fibrillation, arterial aneurysms, hypercoagulable states, and iatrogenic thromboembolism [1]. The typical presentation of ALI symptoms is the "six P's": pain, pulse deficit, paralysis, pallor, poikilothermia, and paresthesia [1]. The diagnostic tools include duplex ultrasound (DUS), computed tomography angiography (CTA), and magnetic resonance angiography (MRA) [3]. ALI has a relatively poor prognosis, with the sudden loss of limb function resulting in the amputation of the affected limb [1]. Vascular medicine, vascular surgery, and interventional therapy are the key to prompt revascularization in ALI [3]. Treatment begins with intravenous (IV) unfractionated heparin infusion to prevent thrombus progression within the rest of the limb [3]. The signs of cyanosis, paralysis, and stiffness indicate irreversible ischemia, and urgent amputation is needed in such cases [3]. Treatments include a surgical approach or endovascular management. Surgical approaches include thromboembolectomy and bypass, while endovascular approaches include catheter-directed thrombolysis (CDT) and stent placement. Sometimes a combination of the two methods is used [1]. 

Heparin-induced thrombocytopenia (HIT) is a hypercoagulable state that can develop immune-mediated reactions against any form of heparin products [4]. It can be characterized into type one HIT (non-immune mediated) and type two HIT (immune-mediated) [5]. Type two HIT is a more common condition due to heparin's antibody-mediated reaction to platelet factor four (PF4), leading to thromboembolic complications such as deep venous thrombosis and pulmonary embolism [4]. The most apparent outcome is thrombocytopenia caused by consumption of IgG-coated platelets by macrophages and the reticuloendothelial system [4]. HIT management involves immediate discontinuation of heparin and switching to argatroban, bivalirudin, danaparoid, fondaparinux, or a direct oral anticoagulant (DOAC) [5].

Coronavirus disease 2019 (COVID-19) is the global pandemic known mainly for acute respiratory distress syndrome [6]. However, studies have reported that COVID-19 has severely impacted various integrated body systems, including the coagulation parameters [6-8]. The deranged coagulation parameters create a hypercoagulable state, increasing the risk for a thromboembolic event [6-10]. Recent studies of peripheral arterial involvement have also been published that correlate COVID-19 to incidences of ALI [7-10]. 

We present a case of a patient with an asymptomatic COVID-19 infection who suddenly suffered from ALI, which was further complicated by HIT and other clinical implications.

## Case presentation

This report involves a 49-year-old Caucasian male with no significant past medical history who presented to the emergency department (ED) complaining of pain and coldness in the right foot with some tingling for the past few hours. The pain was described as vague and had a progressive onset. The patient has a history of smoking one pack per day for the last 30 years, and he works as a general contractor. At the time of presentation, the patient was able to move his toes and foot without much difficulty. The right foot examination revealed diminished dorsalis pedis pulse, mild sensory loss, and no muscle weakness. The right foot was cold and displayed pallor with no distinct demarcation. The patient had a CTA done, which revealed an acute occlusion involving the proximal anterior tibial artery and the tibioperoneal trunk with reconstitution distally. Given the poor reconstitution, no evidence of atherosclerotic disease in either extremity and no evidence of vasculitis the occlusions were likely acute and embolic in etiology. The patient was admitted to the intensive care unit (ICU), a tissue plasminogen activator (TPA) was infused directly into the affected peripheral vessel, and a heparin drip was started.

**Figure 1:**
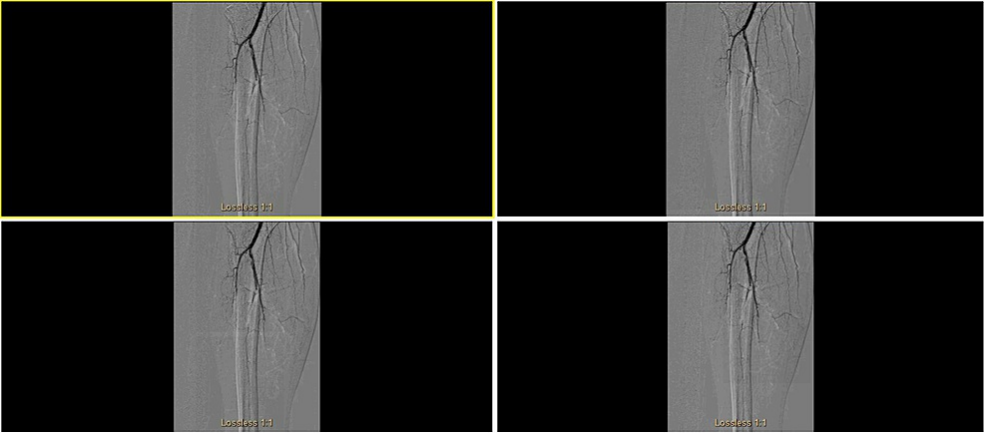
Percutaneous transluminal angiogram of the right leg tibial arteries demonstrating blocked tibial vessel.

The patient did not have any history of cardiac arrhythmias or myocardial infarction. An echocardiogram revealed a patent foramen ovale which was only evident by a bubble study. Due to the foramen ovale not being large enough to explain the current arterial occlusion, a transesophageal echocardiogram and a closure device were not recommended. Interventional radiology was consulted, and the patient underwent four attempts of thrombolysis, resulting in improved clot burden by the end of each procedure. However, the patient continued to have repeated episodes of thrombosis. Angioplasty of the anterior tibial and posterior tibial arteries was also performed. Unfortunately, recanalization of these vessels was unsuccessful. Despite four days of TPA thrombolysis therapy and multiple attempts at angioplasty and thrombectomy of all three tibial vessels, they remained occluded. The patient's right lower extremity angiogram demonstrated persistent occlusion of all three tibial vessels below the knee in concurrent images (Figure 1). There was also rethrombosis of the peroneal artery and anterior tibial artery.

Hypercoagulable workup was negative for factor V Leiden, lupus, antiphospholipid syndrome, and vasculitis. Furthermore, the activity of protein C, protein S, and anti-thrombin III was normal. Seven days after the patient's initial presentation, he had worsening discoloration, advancing pain, and a cold avascular foot, ankle, and calf. The patient's foot was insensate, with the inability to move toes, severe mottling, and darkening of the skin below the ankle. There was also mottling of the distal third of the leg. A right below-knee amputation was planned to prevent infectious gangrene. At the time of amputation, the patient was noted to have avascular gangrene of the distal half of his right lower extremity, especially lateral and anterior compartments, with non-contractile muscle. Complete occlusion of all arterial portions of the vascular tree was found during amputation, with a gelatinous clot. Pathology reports stated findings of an embolic clot with some atherosclerotic changes in the posterior tibial vessels.

Postoperative D-dimer was elevated at 12,021 ng/mL FEU and platelets dropped from 253,002 to 26,000 in two days. HIT was suspected which is a clinicopathologic diagnosis that requires combined evaluation of clinical examination and laboratory test results. The 4Ts scoring system was used to support the need for laboratory testing (Table 1). This case resulted in a score of at least 6 which is correlated with a high probability of HIT. Heparin was held, and HIT testing with a serotonin release assay was positive. Therefore, he was started on an argatroban drip and coumadin. The argatroban drip was stopped once his INR was therapeutic.

**Table 1:** 4Ts scoring system * A score is assigned for each category or variable, and the total 4Ts score (far right column) is the sum of all scores. IV: intravenous

4Ts Scoring System					
Score*	Category or Variable				Total Score
	Thrombocytopenia	Timing of Onset	Thrombosis	Other Causes of Thrombocytopenia	
2	Platelet count decreases >50% Platelet nadir ≥20 x 10^9^/L	5-10 days after start of heparin, or ≤1 day (previous heparin exposure within 30 days)	New thrombosis, or skin necrosis at heparin injection sites, or acute systemic reaction after IV heparin	No other apparent cause	6-8 (high)
1	Platelet count decreases 30-50% Platelet nadir 10-19 x 10^9^/L	>10 days after start of heparin, or time frame of onset is unclear	Progressive or recurrent thrombosis	Possible other cause	4-5 (intermediate)
0	Platelet count decreases <30% Platelet nadir <10 x 10^9^/L	≤4 days after the start of heparin, with no recent heparin exposure	No thrombosis	Definite other cause	0-3 (low)

COVID-19 IgG and IgM antibody tests were positive even though testing at the time of admission via nasal swab, reverse transcription-polymerase chain reaction, and nucleic acid amplification were negative. This raised a concern that the clotting was possibly a complication of a previous COVID-19 infection, although the patient denied any history of COVID-19 infection and showed no other symptoms.

**Figure 2:**
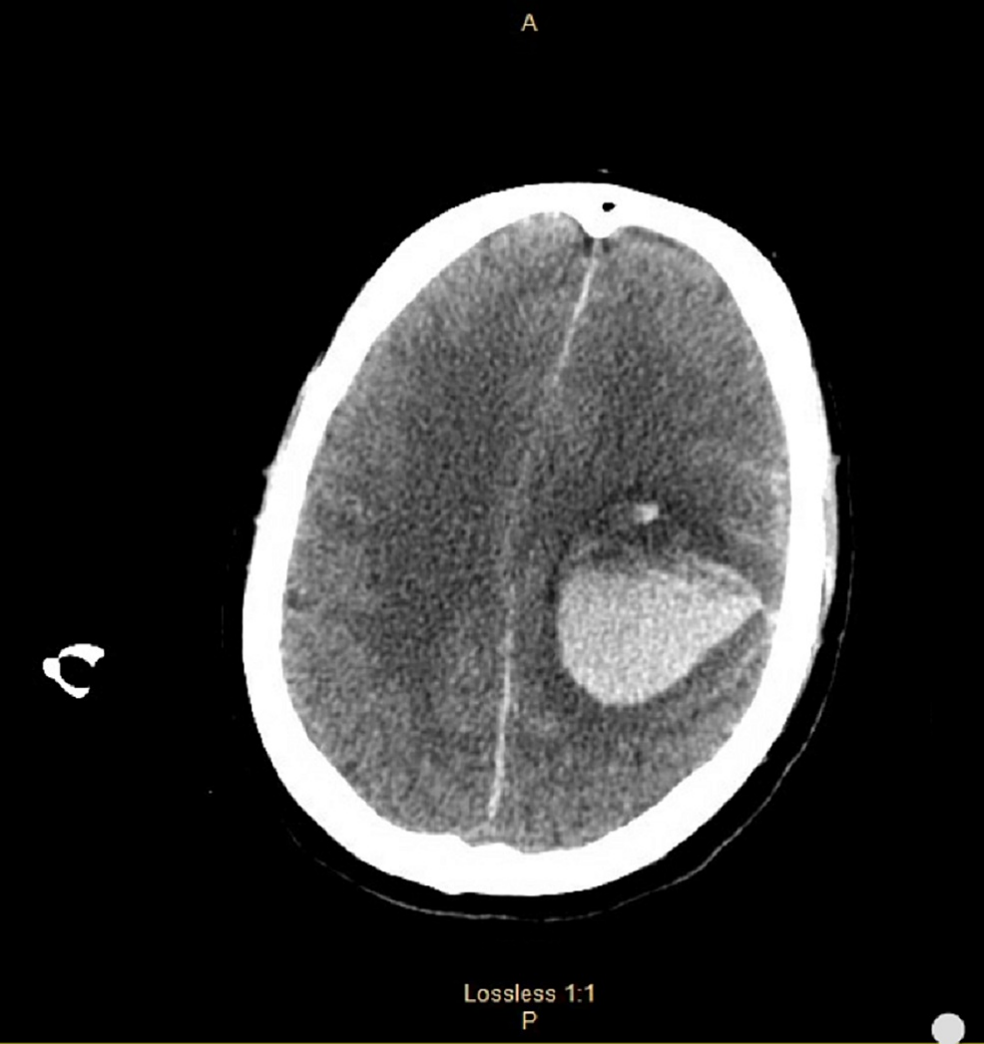
CT of the head without contrast showing extensive intraperitoneal hemorrhage within the left frontoparietal region with extension into the cerebral ventricles.

Once stabilized, the patient was discharged on 6 milligrams of warfarin and low-dose aspirin daily. After discharge, the patient was lost to follow up and was brought to the ED 13 days later by ambulance complaining of severe headache and altered mental status. When the patient arrived at the ED, he was minimally responsive and appeared lethargic with no apparent trauma. The patient was not responding to painful stimuli, no posturing was noted, and his pupils were reactive to light and measured at 4 millimeters. CT of his head showed massive intraparenchymal and intraventricular hemorrhages with extension into the third ventricle and a rightward shift consistent with subfalcian herniation as well as downward herniation (Figure 2).

The patient decompensated, his respiratory efforts became closer to agonal, and the patient was intubated. The patient's INR was 7.2. He was given fresh frozen plasma, vitamin K, and Kcentra. The patient was transferred to another hospital better equipped to handle critical patients but could not survive.

## Discussion

**Figure 3:**
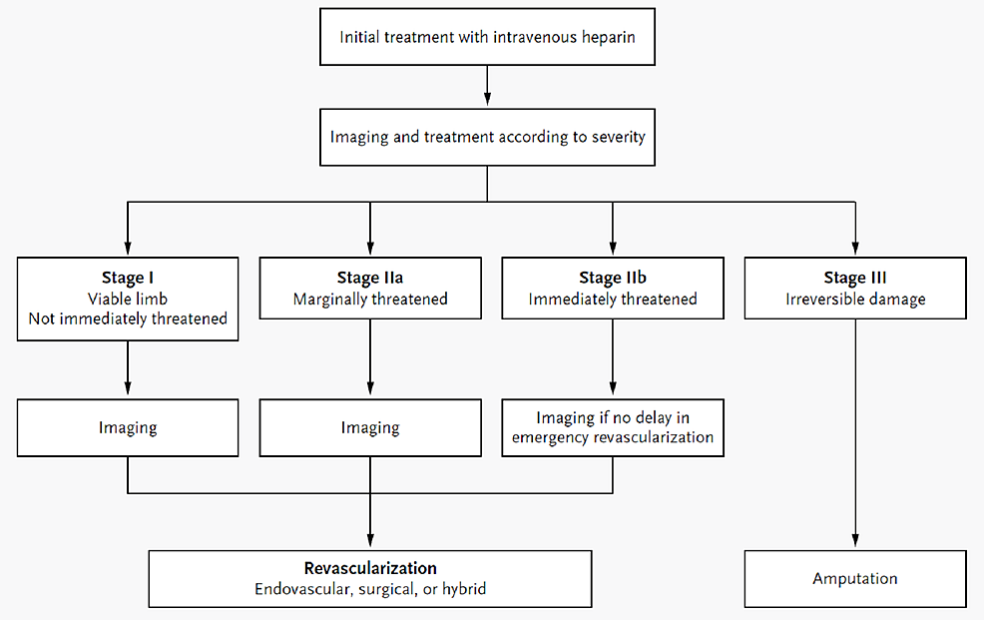
Algorithm for the treatment and diagnosis of acute limb ischemia

ALI presents with sudden and severe symptoms within two weeks. This is due to the lack of development of collateral circulation, which is in contrast with chronic ischemic conditions [3]. The severity of ALI is categorized using the Rutherford classifications. It is based on the clinical presentation, including sensory and motor findings and both arterial and venous Doppler signals (Table 2) [11]. Based on these classifications, the diagnosis and treatment method is determined (Figure 3) [12].

**Table 2:** Stages of acute limb ischemia

		I	IIa	IIb	III
Classification		Viable	Marginally Threatened	Immediately Threatened	Irreversible
Description		Not immediately threatened	Salvageable if treated promptly	Salvageable with immediate revascularization	Inevitable permanent nerve damage or major tissue loss
Findings	Sensory	None	Minimal (toes) or none	Associated with rest pain; more than toes	Anesthetic; Profound
	Motor	None	None	Mild to moderate	Profound; paralysis (rigor)
Doppler Signals	Arterial	Audible	Often inaudible	Usually inaudible	Inaudible
	Venous	Audible	Audible	Audible	Inaudible

Based on the patient's presentation of acute onset pain, tingling, and coldness of the right foot, the patient's ischemia of the lower extremity was likely classified into Rutherford category two, known as threatened. Hence, instead of doppler, the patient underwent a CTA, which was consistent with ALI findings. Despite appropriate treatment, the patient's right lower extremity ischemia worsened within four days, progressing from Rutherford classification two to classification three consistent with irreversible ischemia. Subsequently, the extremity had to be amputated to prevent infectious gangrene. Moreover, the platelet count dropped significantly within two days making the diagnosis consistent with HIT. Thrombosis due to HIT occurs via platelet activation and endothelial cell injury. Both venous and arterial thrombi are associated with HIT, with venous thrombi much more common. Venous thrombotic complications include limb gangrene and pulmonary embolism [13], whereas arterial thrombosis can lead to stroke, myocardial infarction, ALI, and organ infarction [14]. Typically, HIT develops five to ten days after the initiation of heparin [15]. Nevertheless, early onset may be seen in patients who already have circulating HIT antibodies due to previous heparin exposure [16].

The common hemostasis abnormalities related to COVID-19 infection are thrombocytopenia and elevated D-dimer, as seen in our patient [17]. This abnormal hemostasis increases the risk of thromboembolic disease, respiratory failure, or death [17,18]. The disease severity is associated with prolonged prothrombin time (PT), INR, and thrombin time (TT), and decreased activated partial thromboplastin time (aPTT) [17]. A recent study found that a higher D-dimer, fibrin degradation products, and prolonged PT were seen in patients with COVID-19 infection who died compared to those who survived [6]. However, it is still unknown if these hemostatic changes are caused explicitly by COVID-19 infection or precipitated by cytokine release from systemic inflammatory response syndrome [17]. Hospitalized patients with severe acute respiratory syndrome coronavirus 2 (SARS-CoV-2), especially patients with comorbid conditions, should receive prophylactic anticoagulation to prevent thromboembolic disease and further complications [6,17-18]. The recommended prophylaxis by the World Health Organization (WHO) is daily low-molecular-weight heparins, or twice-daily subcutaneous unfractionated heparin (UFH) [17]. Although our patient was on IV heparin for more than one week, his clinical deterioration was owing to concurrent HIT.

This case highlights the importance of diagnoses and treatment of ALI promptly. The patient does not have a history of significant cardiovascular disease and did not present any symptoms of COVID-19 infection. Regardless of the patient's past medical history and current health condition, prophylactic anticoagulation should be started immediately. It is also crucial to perform appropriate lab testing regularly. If the patient does not have clinical improvement within 24-48 hours, physicians should suspect potential HIT and confirm the diagnosis. This will prevent complications such as loss of complete blood supply and infectious gangrene requiring amputation. Additionally, physicians should be aware of life-threatening hemorrhage when a patient is on any anticoagulant medication. Regular follow-up and prompt management are the keys to patient survival.

## Conclusions

This case reports an asymptomatic COVID-19 patient with no significant comorbidities who suffered from ALI complicated by HIT. Vascular emergencies such as sudden loss or marked decrease in limb perfusions consistent with ALI need to be diagnosed and treated promptly. The key to prompt revascularization in ALI is vascular medicine, vascular surgery, and interventional therapy. Prophylactic anticoagulation should be initiated immediately, as long as the patient is not currently bleeding. Monitoring regular lab tests while the patient is on anticoagulation should be done to detect complications such as HIT early. Patients should be educated on the risks of anticoagulation therapy as life-threatening complications such as hemorrhage can occur. Therefore, patient compliance is a significant factor in the appropriate management of the disease. Finally, when individual patient needs are addressed, a multidisciplinary method will provide a stronger outcome and quality of life.
